# Cohort Profile: The Norwegian Life Course, Ageing and Generation Study (NorLAG)

**DOI:** 10.1093/ije/dyaa280

**Published:** 2021-01-26

**Authors:** Marijke Veenstra, Katharina Herlofson, Marja Aartsen, Thomas Hansen, Tale Hellevik, Gry Henriksen, Gøril Kvamme Løset, Hanna Vangen

**Affiliations:** 1 Norwegian Social Research (NOVA), Oslo Metropolitan University, Oslo, Norway; 2 Norwegian Centre for Research Data, Bergen, Norway

**Key Messages**

- The Norwegian Life Course, Ageing and Generation Study (NorLAG) was set up to gain new and updated knowledge on ageing and age-related changes in Norway.
- The nationwide, population-based study includes information on core life domains for 11 028 men and women born between 1922 and 1966. NorLAG combines longitudinal survey data from three waves (2002, 2007 and 2017) with secondary annual data from the public registers that provide time series on financial information, civil status and educational attainment for up to 50 years (1967 to 2017).
- NorLAG1 comprises 5555 respondents aged 40 years and older at the time of interview, the expanded NorLAG2 sample counts 9238 respondents (including 68% of the NorLAG1 participants) and NorLAG3 includes a total of 6099 respondents (aged 50–95).
- Topics covered include health and care, wellbeing and mastery, work and retirement, and family and intergenerational relationships. Information on context, timing of events and close family enables the construction of interdependent trajectories and pathways for men and women from mid-life to old age.
- Access to the NorLAG data is facilitated through the national research infrastructure ACCESS Life Course. Data are available via the online portal at [https://norlag.nsd.no], hosted by Norwegian Social Research at Oslo Metropolitan University and the Norwegian Centre for Research data.

## Why was the cohort set up?

The Norwegian Life Course, Ageing and Generation Study (NorLAG) was launched in 2002 in order to gain new and updated knowledge on ageing and age-related changes in Norway in the 21st century. With nationwide longitudinal survey data linked to annual data from public registers, NorLAG provides novel opportunities for scholars to study the prevalence, the timing and sequencing, and the causes and consequences of transitions in individual life courses among men and women aged 40 years and older. The longitudinal design enables researchers to not only examine the various phases in the second half of life, but also to explore ageing as a process which may unfold differently across social groups (e.g. gender, socioeconomic position, birth cohort and local context). A central aim of the study is to strengthen the foundation for empirical ageing and welfare research and to be an important tool in creating new knowledge that can feed into policy goals, such as enhancing active ageing and intergenerational solidarity, and reducing social inequalities across the life course.

The NorLAG study was initiated by Norwegian Social Research (NOVA). The survey data collections conducted so far have been financed by the Research Council of Norway, four Norwegian Government Ministries, the Norwegian Directorate of Health, the Norwegian State Housing Bank, Statistics Norway and NOVA. Data from NorLAG are part of the national ACCESS Life Course Infrastructure, funded through the National Financing Initiative for Research Infrastructures at the Research Council of Norway.^1^

## Who is in the cohort?

NorLAG includes a nationwide, population-based and stratified sample of adults born between 1922 and 1966. Survey data from three waves and annual data from the public registers, comprising 11 028 respondents, are available for research and education. The sample is drawn from the National Population Register, which covers the entire population of Norway, by using national identification numbers. Statistics Norway, the national statistical institute of Norway, has been responsible for sampling procedures and data collections. In the first survey wave (NorLAG1), participants were sampled from the birth cohorts 1922–1961 (aged 40–80 years at the time of interview), living in 24 municipalities and six townships across Norway. Within each municipality and township, the sample was stratified according to gender and age. Data for NorLAG1 were collected during 2002 and 2003. The second wave (NorLAG2) was conducted 5 years later, in 2007–08. The sampling frame included the gross sample of NorLAG1 and was expanded in two ways. First, sampling was no longer restricted to the original 30 municipalities and townships, but included the whole country. The sample was stratified according to gender, age, geographical region and centrality of residential municipality (most central to least central), resulting in 78 strata and with new participants sampled to fill in empty strata. Second, refreshment samples were added, for birth cohorts 1922–1961 in order to compensate for dropout, and by including younger birth cohorts i.e. 1962–1966. The third wave of NorLAG (NorLAG3) was conducted in 2017 and included all living participants born between 1922 and 1966 who had responded to NorLAG1 and/or NorLAG2. At the time of the NorLAG3 interview, the youngest respondents were aged 50 and the oldest 95 years.

Initial contact with eligible participants in NorLAG1 and NorLAG2 consisted of an invitation and an information brochure that were sent by postal services informing about the aim of the study and data collection. In NorLAG3, initial contact was made via e-mail with an information brochure attached. Participants without e-mail information received the invitation and the brochure through the postal service. In each wave, all eligible participants were subsequently contacted by telephone to confirm participation.

**Figure 1:**
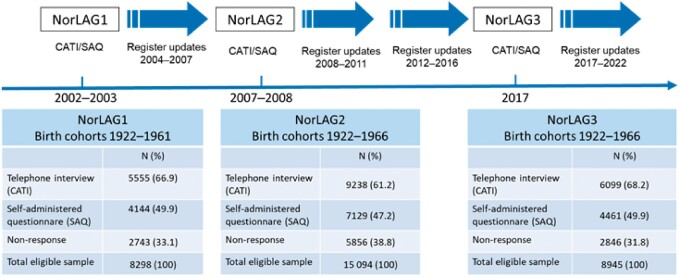
Data collections in the Norwegian Life Course, Ageing and Generation Study (NorLAG). CATI, computer-assisted telephone interview; SAQ, self-administered questionnaire

Response rates in the three consecutive survey waves are 67%, 61% and 68% (Figure 1). Main reasons for non-response in each wave were: refusal to participate (19–27%), non-contact (4–9%) and health or language limitations (4–5%). Loss to follow-up due to mortality in the total sample of participants (*N* = 11 028) corresponded to 1700 persons by 2017. Sampling weights have been calculated for NorLAG2 and NorLAG3. Detailed information for each of the three waves on the sampling design, the calculation of response rates and sampling weights is available in the documentation reports provided by Statistics Norway.^2–4^

NorLAG employs two different interview modes. First, all participants are contacted for a computer-assisted telephone interview (CATI). After completion of the CATI, participants receive a self-administered questionnaire (SAQ). Respectively 75%, 77% and 73% of the CATI respondents have returned the SAQ in the three subsequent waves. In NorLAG3, participants could choose between a web-based and a postal questionnaire. The majority (81%) of the SAQ respondents completed the web-based version. Those opting for the postal version were substantially older (mean age 73 [standard deviation (SD) = 9.4] versus 63 years (SD = 8.3; *P* < 0.0001).

Participation in the NorLAG study is voluntary and based on passive informed consent. All data collections conform to the regulations related to the implementation and supplementation of official statistics,^5^ and are in line with the regulations on the processing of personal data. To ensure and protect anonymity of the respondents, personal identifiers are replaced with unique anonymous identifiers, and detailed information on income, education, profession, welfare benefits and medical (self-reported) diagnoses are grouped into larger categories.

Table 1 provides comparisons of sociodemographic characteristics (gender, age, educational attainment) of the NorLAG participants with corresponding distributions for the Norwegian population at the time of sampling. The NorLAG samples represent the general Norwegian population well when it comes to age and gender. Across all three waves, people with basic education are, however, under-represented in the study, whereas those with high education (university college or higher) are over-represented.

**Table 1:** Samples in he Norwegian Life Course, Ageing and Generation Study (NorLAG) compared with the total Norwegian population by survey year, sex, age at time of interview and level of education

Data collection	2002–03			2007–08				2017			
	*N*	%	% of total population (31.12.2001)	*N*	%	Valid % on the full weighted sample^a^	% of total population (31.12.2006)	*N*	%	Valid % on the full weighted sample	% of total population (31.12.2016)
Men	2699	48.6	48.0	4532	49.1	49.7	48.1	3013	49.4	48.6	48.5
Women	2856	51.4	52.0	4706	50.9	50.3	51.9	3086	50.6	51.4	51.5
Age groups^b^											
40-49	1546	27.8	30.8	2659	28.8	31.0	30.4				
50-59	1656	29.8	28.0	2683	29	30.1	27.6	2026	33.2	34.8	36.4
60-69	1254	22.6	17.4	2282	24.7	23.4	20.3	2071	34.0	31.3	31.0
70-79	1030	18.5	15.2	1284	13.9	14.7	13.1	1428	23.4	21.3	20.6
80-95	69	1.2	8.5^c^	330	3.6	n.a.	8.5^c^	574	9.4	12.6	12.0 ^c^
Level of education^d^											
Basic	1477	26.7	34.1	2031	21.2	28.4	29.1	954	15.7	26.4	26.6
Secondary	2639	47.7	45.1	4643	48.4	47.0	46.3	2861	47.1	46.7	46.5
Higher	1419	25.6	20.9	2926	30.5	24.6	24.6	2257	37.2	26.9	26.8

a Sampling weights NorLAG2 are calculated for sample 40–79 years.

b Age groups refer to age at time of computer-assisted telephone interview (CATI).

c Statistics refer to age group 80–89 years.

d Statistics for national population refer to age group 40+ in NorLAG1 and 2; 50–95 in NorLAG3.

n.a. , not applicable.

## How often have participants been followed up?

So far, three waves of survey data collection have been carried out (2002, 2007 and 2017). Two additional waves are planned for, with a fourth wave scheduled to take place in 2023. Primary data sources are survey data consisting of CATI and a subsequent SAQ sent to the respondents within 1 week after the telephone interview. The mean length of the CATI was 32 min in the first wave, 43 min in the second and 30 min in the third wave of NorLAG. The CATI interview uses information from the registers and available data from previous interviews to ease the burden on respondents. Together with complex routing structures this procedure leads to compound file structures, with varying interview lengths depending upon the life situation of the respondents. The SAQ consists of 20 pages (postal version) and 40 (batteries of) questions.

Secondary sources for data collection are administrative register data including the Population register, the Tax and Income register, the National database on Education, the Birth register, and Statistics Norway’s historical events database (FD-Trygd). Register data have been used for sampling and data quality control by Statistics Norway, as well as for supplemental data sources before and after the CATI. Annual updates of the registers are available for respondents up to 5 years after each survey wave. Updated retrospective register data have been added for all NorLAG3 respondents. This information provides time series on income and financial information, sickness absences, civil status and educational attainment for up to 50 years (1967 to 2017). For NorLAG2 and NorLAG3 participants, register data are also available for the respondents’ partners.

Baseline and follow-up characteristics are provided for two complementary but distinct samples of NorLAG: (i) the NorLAG1 cohort of 8298 eligible individuals (born 1922–61 and selected from 30 municipalities and townships); and (ii) the NorLAG2 cohort, a national representative eligible sample of 15 094 persons (born 1922–66). Note that the sample of NorLAG3 is contingent upon participation in one of the two previous waves.

Characteristics of the NorLAG1 cohort are given in Table 2 for each of the three waves, as well as for the three-wave participants. Level of education and self-rated health are strongly related to study retention: respondents with higher education and good/excellent self-rated health are more inclined to participate in all three waves. Loss to follow-up due to mortality in the NorLAG1 cohort (*N* = 1352) is higher among older people, men, participants with basic compulsory education, and those with fair/poor self-rated health (see Table 2). All in all, 76% (*N* = 6292) of the NorLAG1 cohort participated at least once, 4456 persons participated at least twice and 1836 persons participated only once. Considering only respondents participating at baseline (*N *= 5555), 3765 (68%) re-participated in NorLAG2 and 2330 (42%) responded to all three waves.

**Table 2:** Sociodemographic and health characteristics of the two cohorts of The Norwegian Life Course, Ageing and Generation Study (NorLAG); NorLAG1 Cohort (left) and the NorLAG2 Cohort (right)

	NorLAG1 cohort						NorLAG2 cohort			
	NorLAG1 (2002–03)	NorLAG2 (2007–08)	NorLAG3 (2017)	NorLAG12	NorLAG123	Deceased (per March 2017)	NorLAG2 (2007–08)	NorLAG3 (2017)	NorLAG23	Deceased (per March 2017)
	*N *= 5555	*N *= 4502	*N* = 3021	*N* = 3765	*N* = 2330	***N* = 1352** ^c^	*N* = 9238	*N* = 6099	*N* = 5711	N = 1005^d^
Gender (%)										
Men	48.6	48.5	48.8	48.7	48.7	55.3	49.1	49.4	49.5	57.7
Women	51.4	51.5	51.2	51.3	51.3	44.7	50.9	50.6	50.5	42.3
Education (%)										
Compulsory	26.6	21.9	17.1	20.9^a^	16.0^a^	37.9^a^	20.8	15.7	15.4	32.6
Secondary	47.5	47.9	47.3	48.0	47.6	46.6	48.2	46.9	47.1	48.2
Tertiary	25.5	29.9	35.0	30.8	36.3	15.0	30.7	37.0	37.3	18.7
Missing	0.4	0.3	0.5	0.3	0.1	0.4	0.3	0.4	0.2	0.5
Living with partner (%)										
No	30.1	30.9	34.3	27.5^b^	24.0^b^	45.1^b^	27.2	30.9	23.3^e^	44.5^e^
Yes	69.9	69.1	65.7	72.5	76.0	54.9	72.8	69.1	76.7	55.5
Self-rated health (%)										
Good-excellent	72.6	69.6	68.9	77.0^b^	81.6^b^	57.2^b^	72.2	71.1	77.4^e^	53.2^e^
Fair-poor	27.4	30.0	30.9	23.0	18.4	42.8	27.4	28.6	22.3	45.9
Missing	0.0	0.4	0.2	0.0	0.0	0.0	0.4	0.3	0.3	0.9

a Age/education in 2002.

b Value at baseline (NorLAG1).

c 1216 of NorLAG1 respondents are deceased.

d *N* = 1005 refers to mortality conditional on participation in NorLAG2.

e Corresponding to values at NorLAG2 (2007).

Table 2 also shows the characteristics of the NorLAG2 cohort, which consists of the gross sample of the NorLAG1 cohort and the large refreshment sample. A total of 9238 persons participated in NorLAG2, of whom 62% (*N* = 5711) responded to NorLAG3 and 11% (*N* = 1005) had died by 2017. Patterns characterizing study retention and loss to follow-up due to mortality are similar to those for the NorLAG1 cohort.

## What has been measured?

NorLAG has a broad multidimensional approach to studying ageing and later life, by exploring stability and change in inter-related major life domains. The four main domains are: (i) Work and retirement, (ii) Family and intergenerational relationships, (iii) Well-being and mastery and (iv) Health and care. The linkage of all three data sources (CATI, SAQ and registers) provides comprehensive information on all topics. In addition, NorLAG includes information on the respondent’s close family members (i.e. partner, parents and children). An overview of the content of the database is provided in Table 3.

**Table 3:** Overview over modules and measures in the three waves of data collection in the Norwegian Life Course, Ageing and Generation Study (NorLAG)

	NorLAG1	NorLAG2	NorLAG3
	**2002**–**03**	**2007**–**08**	2017
Key variables			
Year and month of interview, wave participation, mode participation, sampling weights	CATI/SAQ	CATI/SAQ	CATI/SAQ
Sociodemographics respondent (age, birth year, gender, year of death)	Register	Register	Register
Immigration background	Register	Register	Register
Education respondent/partner (level and subject) 1978-2017^a^	Register	Register	Register
Work and retirement			
Employment of respondent (type of occupation, working hours, work leave, job characteristics, job satisfaction)	CATI	CATI	CATI
Retirement planning and motivations	CATI	CATI	CATI
Psychosocial work environment	CATI	CATI	CATI
Causes of retirement	CATI	CATI	CATI
Sickness absence of respondent/partner 1992-2017		Register	Register
Income and wealth			
Pensionable income of respondent/partner (1967-2017)			Register
Income, wealth, social benefits, debts respondent/partner (2002-17)^a^	Register	Register	Register
Family and intergenerational relationships			
Siblings, grandparents, friends	CATI	CATI	CATI
Perceived relationship quality	SAQ	SAQ	SAQ
Support exchanges (emotional, instrumental, financial)	CATI	CATI	CATI
Help with child care		CATI	
Children			
Sociodemographics children/stepchildren (age, gender)	Register	Register	Register
Partner			
Partner status, age of partner	CATI+Register	CATI+Register	CATI+Register
Partner history		CATI	CATI
Employment of partner (type of occupation, working hours, work leave, job characteristics)		CATI	CATI
Sharing household tasks, relationship quality		CATI	CATI
Parents			
Parents’ year of birth/death, marital status, education	CATI+Register	CATI+Register	CATI+Register
Parents’ health and care needs		CATI	CATI
Contact with parents, perceived relationship quality	CATI	CATI	CATI
Household box (sociodemographics partner, children living with respondent)	Register and CATI	Register and CATI	Register and CATI
Civil status of respondent 1978-2017	Register	Register	Register
Wellbeing and mastery			
Anxiety and depression: SCL-5^6^ and CES-D^7^	SAQ	SAQ	SAQ
Satisfaction with life,^8^ domain-specific satisfaction	SAQ	SAQ	SAQ
Positive-negative affect (PANAS)^9^	SAQ	SAQ	SAQ
The Short Loneliness Scale^10^		CATI	
Single items loneliness	SAQ/CATI	SAQ/CATI	SAQ/CATI
Personality characteristics			
Big Five Inventory,^11^ BEM sex roles,^12^ Self-Esteem Scale,^13^ Self-efficacy^14^	SAQ	SAQ	SAQ
Locus of control, the Psychological Well-Being Scale	SAQ		
Perceived age-related changes, subjective age	SAQ	SAQ	SAQ
Personal Mastery Scale^15^	CATI/SAQ	CATI/SAQ	CATI/SAQ
Health and care			
Longstanding health problem, longstanding health limitations, (Instrumental) Activities of Daily Living	CATI	CATI	CATI
Self-rated health: Short Form 12^16^	CATI	CATI	CATI
Sensory functions and walking impairments	CATI	CATI	CATI
Lifestyle factors (smoking, alcohol consumption, physical activity, body mass index)	SAQ	SAQ	SAQ
Medication use and health care use	SAQ	SAQ	SAQ
Longstanding health limitations, memory problems and care needs for household members and parents	CATI	CATI	CATI
Professional health care (respondent, household members, parents)		CATI	CATI
Informal care		CATI	CATI
Home and environment			
Housing, neighbourhood	CATI	CATI	CATI
Region/urban/rural	register	register	register
Mortgage, material assets, pets	CATI	CATI	CATI
Values and attitudes			
Religion, religiosity, political party	CATI	CATI	CATI
Attitudes towards sick leave			SAQ
Subjective life expectancy			CATI
Attitudes towards own ageing, sexual orientation	SAQ	SAQ	SAQ
Filial responsibility/attitudes towards welfare state, Basic Human Values Scale^17^	SAQ	SAQ	SAQ
Life events			
Major life events (childhood, adulthood, recent)	SAQ	SAQ	SAQ
Activities			
Leisure activities	SAQ	SAQ	SAQ
Volunteering			SAQ+CATI

SAQ, Self-Administered Questionnaire; CATI, Computer-Assisted Telephone Interview.

a No register information for partner in NorLAG1.

The choice of survey questions and measures is based on psychometric properties, the relevance for welfare policy and cross-age validity, as well as on more pragmatic factors related to the availability of tools in Norwegian, mode of interview (CATI and SAQ) and limited interview time. With an emphasis on exploring ageing as a process, cross-wave comparability is a priority. Thus, questions and measurement instruments have been kept as stable as possible across waves. Exceptions are: (i) the introduction of new topics or questions of particular relevance at the time of the survey wave (e.g. volunteering and work exits); and (ii) the collaboration between NorLAG and the Generations and Gender Survey^18^ in wave 2. The latter implied, for example, a revision of the response scales for some questions, from a five-point Likert scale (1–5) to an 11–point scale (0–10).

### Work and retirement

Survey questions on work and retirement address aspects of job involvement and job satisfaction, as well as retirement intentions and behaviour and consequences of work exits. Survey responses are linked to extensive annual register data, including various measures of income (from work, social welfare, pensions), sickness absences (days per year) and pension uptake (year and share). All income data are truncated to the nearest 10 000 NOK to secure anonymity.

### Family and intergenerational relationships

NorLAG data provide the opportunity to map family networks in later life and to investigate the potential for solidarity across family generations. Survey questions are inspired by the ‘intergenerational solidarity model’^19^ and include the following dimensions: family structure, contact, relationship quality, help and care, and family responsibility norms. The most extensive battery of questions refers to respondents’ partners, parents and adult children, but questions about siblings, grandparents, grandchildren and stepchildren are also included.

### Well-being and mastery

Longer lives have contributed to an increased policy interest in adding quality to years of life, and in the related concepts of mental health, successful and active ageing and ageing well. NorLAG comprises a wide variety of validated measures and scales on well-being and mastery, covering cognitive (life satisfaction, domain-specific satisfaction), affective (positive and negative affect) and eudemonic (self-esteem, sense of control) dimensions. The surveys also include questions on loneliness and validated instruments on mental health (e.g. depression and anxiety).

### Health and care

Investigating factors that contribute to maintaining good health and functioning in old age (healthy ageing) and assessing care needs in an ageing society are important objectives within the NorLAG study. The three waves of survey data collection include a broad range of self-report health measures, including chronic conditions, functional health status, (instrumental) activities of daily living, body mass index and sensory functioning (vision and hearing). Information on self-reported health conditions has been re-coded into ICD-10 codes and, to secure anonymity, grouped into ICD-10 chapters. Health behaviour is measured mainly through questions in the SAQs. The surveys also include questions on health problems of the partners and parents and on formal and informal care exchanges. Information on mortality (death year) stems from the public registers and is currently updated until 2017.

### Additional topics

In addition to the main domains mentioned above, NorLAG also covers a wide variety of batteries and questions on housing, neighbourhood, life events, volunteering, leisure activities, personality, and attitudes and values, as well as subjective age and subjective life expectancy.

## What has NorLAG found?

Analyses based on NorLAG data have generated a wide variety of scientific publications, including studies on predictors of healthy ageing,^20–25^ informal caregiving,^26–29^ age-related changes in well-being^30–33^ and social health inequalities.^22^^,^^34–36^ An overview of all peer-reviewed articles published from 2011 onwards is available at [norlag.nsd.no]. The data have also been used for White Papers, official reports, PhD and master’s theses and for educational purposes. Below we have highlighted some examples of recently published research that can be considered most relevant for the readers of the current journal.

Brunborg^20^ showed that a high level of negative affect at baseline was associated with a considerably greater risk of heavy drinking 5 years later. Thus, similar to what has been found in studies on alcohol consumption among young people, older adults may drink alcohol to reduce negative emotions or to cope with negative thoughts and emotions. In a study on retirement, Grøtting and Lillebø^22^ found a positive association of retirement with physical health, particularly among individuals with low socioeconomic status. This finding underlines the importance of assessing the potential heterogeneity in health effects of retirement across social groups.

**Figure 2:**
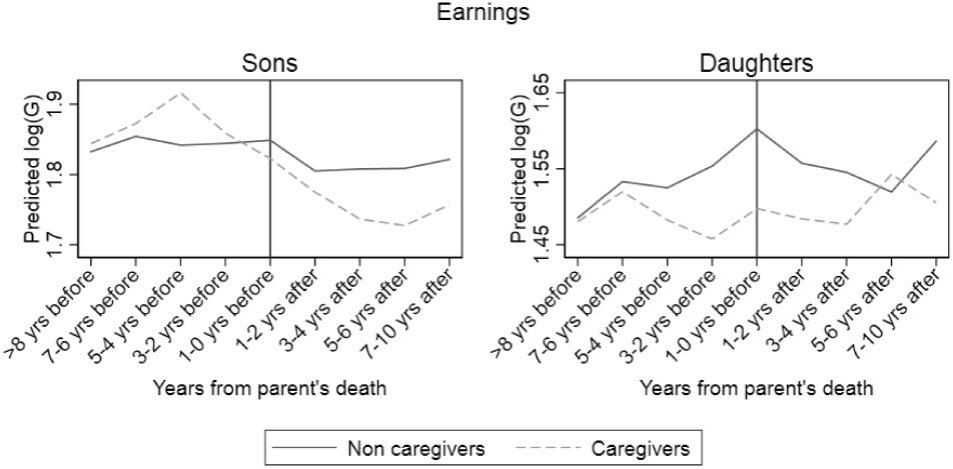
Estimated impact of time (years) to loss of lone parent on the log of standardized earnings (G) for caregivers and non-caregivers, for sons and daughters separately. The figure originates from the following publication: Vangen H (2020)^29^

In a recent study on informal caregiving, Vangen^29^ analysed whether caregivers deviate from non-caregivers in employment and earnings before and after the loss of a lone parent. Using survey data on provision of help and care to parents combined with annual register data on earnings, the author concludes that caring for older parents is negatively related to adult children’s earnings. This negative association is found not only in the year(s) with substantial caregiving needs, but also in the period following parents’ death (see Figure 2).

Findings from a recent study on age-related changes in loneliness^30^ illustrate how age trends vary by gender. Among women, loneliness increases steadily from age 40 to 80, whereas the trend for men follows a U-shaped curve, with highest loneliness levels at age 40 and 80 and lower levels in between. A final example is a study from Enroth and colleagues,^35^ which exemplifies possibilities for cross-national analyses by combining results from NorLAG, the Danish Longitudinal Study of Ageing, the Swedish Panel Study of Living Conditions of the Oldest Old, and the Finnish Vitality 90+ Study. The authors address social health inequalities in old age by assessing self-rated health, mobility and activities of daily living according to level of education among three age groups (75–84, 85–94 and 95+). With only few exceptions, in all age groups, individuals with higher education have more favourable health and functioning compared with those with basic education. The findings show remarkable persistence of health inequalities in the Nordic countries throughout later life.

## What are the main strengths and weaknesses?

The main strengths of NorLAG are the extensive combinations of longitudinal survey and register data, enabling detailed analysis of role changes and life events, such as onset of chronic illness, retirement, partner loss and sickness leave, and their consequences over time.

Survey data complement the register data in providing a broad range of subjective information relevant to respondents’ living conditions and well-being. The inclusion of information about the respondents’ immediate family enables analyses of linked lives, a core principle in life course research. Moreover, the relatively large samples facilitate analyses across and within different social groups (e.g. gender, age group, educational level, employment status). The rich sets of validated health, psychological and sociological scales strengthen the quality of the study and enable cross-national comparative analyses with similar ageing surveys, such as the Survey of Health, Aging and Retirement in Europe (SHARE), the Longitudinal Aging Study Amsterdam (LASA) and the Swedish Panel Study of Living Conditions of the Oldest Old (SWEOLD). An additional strength is that NorLAG is part of a national research infrastructure,^1^ which facilitates secure access to high quality data and this way contributes to increased transparency in the research process, less duplication of research and increased data sharing.

Similar to other longitudinal surveys, and to ageing surveys in particular, a main challenge is related to attrition between waves. Researchers need to address the relevance and extent of selective attrition in relation to the aim of their publications. Although some research has shown that attrition tends to be highest in the first follow-up and attenuates in subsequent ones, avoiding additional attrition in future waves is essential. For cross-sectional analyses, the availability of sampling weights facilitates analyses of separate survey waves.

In longitudinal surveys, it is important to secure continuation of core items and instruments in order to avoid methodological difficulties in analysing changes over time. Questions and measurement instruments in NorLAG have thus been kept as stable as possible across waves, at the expense of possibilities for keeping up with new and perhaps improved measurement techniques. In addition, there is a trade-off between the advantages of a broad large-scale ageing study like NorLAG, and thematic studies that enable more detailed investigation of single trajectories.

As survey data constitute the main data source, most measures in NorLAG are self-reported and thus prone to measurement error and misclassification. The use of established validated scales, SAQ for potentially sensitive questions and linkages to objective register data may reduce this risk. Another potential limitation is the relatively long time span between subsequent waves of survey data collection (5 and 10 years), which may be too long to establish a sequence of events. For several topics, such as family life, work and retirement, the long time span is to some extent compensated by the inclusion of annual register data.

## How can I get hold of the NorLAG data?

An overview of the available NorLAG data and how to gain access to these is available at the dedicated online portal [https://norlag.nsd.no/] for the research infrastructure. The existence of multiple time points, different data sources (i.e. CATI, SAQ and register data) and the extensive use of filter items and ‘loops’ in the NorLAG study, yield complex data that pose substantial barriers for users. To address such barriers, a national research infrastructure (ACCESS Life Course), hosted by Norwegian Social Research (NOVA) at Oslo Metropolitan University and the Norwegian Centre for Research Data (NSD), is being developed to facilitate the required extensive data management and to provide access to the data through new systems that are flexible, secure and user-friendly. The infrastructure is in line with the FAIR principles^37^ and enables easier access to anonymous NorLAG data for non-profit research and teaching purposes.

NorLAG data have been organized in sets of inter-operable modules, labelled with separate prefixes, which can be ordered in several main software formats (e.g. SPSS, STATA). The data structures enable easy conversion between long and wide formats. The online portal includes documentation of NorLAG’s metadata in line with standards from the Data Documentation Initiative and makes use of a thesaurus (ELSST).^38^ All NorLAG data are available through the portal; however, the establishment of the infrastructure and its online portal is still under construction (until 2022). Further development will include possibilities for online data analyses, updated and expanded documentation and the addition of new register and survey data.

Data access is based on a clear procedure for authentication and authorization. Users are assigned a user ID and have to sign a user agreement (electronically) to obtain the data. The data have a DOI number^39^ and data users are obliged to cite this number and the acknowledgements when using NorLAG data in their publications. More information about the infrastructure and the NorLAG data can be obtained by contacting the corresponding author, or any of the co-authors.

The NorLAG data collections have been conducted by Statistics Norway in line with existing rules in the Act no.54 of 16 June 1989 relating to official statistics and Statistics Norway.^5^ Study participation and linkages to public register data are based on informed consent. Collected data can only be used for statistical purposes and research. Anonymous data are made available to external users.

## Funding

The NorLAG survey data collections have been financed by the Research Council of Norway, four Norwegian Government Ministries, the Norwegian Directorate of Health, the Norwegian State Housing Bank, Statistics Norway and NOVA at Oslo Metropolitan University. NorLAG data [https://doi.org/10.18712/norlag3_1] are part of the ACCESS Life Course infrastructure funded by the National Financing Initiative for Research Infrastructure at the Research Council of Norway (195403 and 269920).

## Conflict of interest

None to declare for all authors.
